# ECIS Based Electric Fence Method for Measurement of Human Keratinocyte Migration on Different Substrates

**DOI:** 10.3390/bios12050293

**Published:** 2022-05-03

**Authors:** Yu-Han Hung, Wei-Chih Chiu, Shyh-Rong Fuh, Yi-Ting Lai, Tse-Hua Tung, Chun-Chung Huang, Chun-Min Lo

**Affiliations:** 1 Department of Biomedical Engineering, National Yang Ming Chiao Tung University, Taipei 112, Taiwan; yuhanhung.be05@nycu.edu.tw (Y.-H.H.); alan1000224.y@nycu.edu.tw (W.-C.C.); srfuh@ym.edu.tw (S.-R.F.); g39904002@ym.edu.tw (Y.-T.L.); d40004001@ym.edu.tw (T.-H.T.); 2 Department of Aesthetic Medicine, Chen Hsin General Hospital, Taipei 112, Taiwan

**Keywords:** cell adhesion, cell migration, ECIS, electric fence (EF), wound healing assays

## Abstract

Electric Cell-substrate Impedance Sensing (ECIS) is an impedance-based, real-time, and label-free measuring system for monitoring cellular activities in tissue culture. Previously, ECIS wound healing assay has been used to wound cells with high electric current and monitor the subsequent cell migration. In this study, we applied ECIS electric fence (EF) method, an alternative to electrical wounding, to assess the effects of different surface coatings on human keratinocyte (HaCaT) migration. The EF prevents inoculated cells from attaching or migrating to the fenced electrode surface while maintaining the integrity of the surface coating. After the EF is turned off, cells migrate into the cell-free area, and the increase in measured impedance is monitored. We cultured HaCaT cells on gold electrodes without coating or coated with poly-L-lysin (PLL), poly-D-lysine (PDL), or type-I collagen. We quantified migration rates according to the different slopes in the impedance time series. It was observed that either poly-L-lysine (PLL) or poly-D-lysine (PDL) limits cell adhesion and migration rates. Furthermore, the surface charge of the coated substrate in the culture condition positively correlates with the cell adhesion and migration process. Our results indicate that the EF method is useful for determining cell migration rates on specific surface coatings.

## 1. Introduction

Wound healing is a dynamic and complex process that involves various cell types, the extracellular matrix (ECM), and numerous regulating growth factors [1]. Re-epithelialization is an essential component of wound healing and is regulated by the proliferation and migration of keratinocytes [2,3]. During epidermis restoration following a wound, keratinocytes move from the wound edge into the wound bed to cover the denuded wound surface, proliferate, and re-establish the epithelium’s barrier function [4]. In addition, the formation of an appropriate ECM that facilitates keratinocyte migration is necessary for effective re-epithelialization [5]. Accumulating studies have shown that keratinocyte migration dysfunction impairs the epithelialization process and is potentially associated with many types of chronic non-healing wounds [6]. Numerous underlying mechanisms that regulate keratinocyte migration and the dynamics of cell–ECM interaction remain unclear. Developing new experimental approaches to sensitively analyze cell migration in vitro and in vivo is valuable. Benefits such as automation, multiple replications, and high reproducibility will potentially promote wound healing studies and uncover unknown mechanisms.

A commonly used in vitro migration assay to study the re-epithelialization of keratinocytes is the scratch assay [6]. This method creates a physical gap within a cell monolayer by displacing a group of cells with a pipette tip. Cell migration can be monitored microscopically as keratinocytes move from the edge of the newly created gap into the opening area. The migration rate of the cells can be quantified by analyzing the decrease of the gap area at different time points until the wound gap is closed. While this technique is inexpensive and straightforward, one drawback is that the scratched width is often uneven, yielding less reproducible results [7].

A possibility to electrically generate wounded areas with the defined size in a confluent cell layer is to use electric cell-substrate sensing (ECIS) [8]. ECIS is an impedance-based measuring system for quantifying cellular behaviors in tissue culture [9], including dynamics of cell attachment and spreading [10], the barrier function of epithelial layers [11], in vitro toxicology [12], transendothelial invasion of cancer cells [13], and stem cell differentiation [14]. ECIS has also been used as an electric wound healing assay to wound and monitor cell migration [15]. As ECIS assay is generally performed in small electrode wells, with only a small number of cells and chemicals needed for the experiment. ECIS wound healing assay applies high electric current pulses to the cell-covered electrode and causes cell death [8]. A quick drop in barrier resistance is observed when the dead cells detach from the electrode surface. Following this, cells migrate into the wounded area, and the impedance gradually increases and reflects the healing process. This automated assay has the advantage that the wound area is well defined and highly reproducible. However, the incomplete detachment of cell debris from the electrode surface can cause the impedance fail to entirely drop to the cell-free level [15,16]. The main reason is that the cell density is low or the cell monolayer is not formed for certain cell types. Furthermore, after electrical wounding cell remnants on the electrode surface might obstruct or delay cell migration into the wound area.

An alternative ECIS method, the electric fence (EF), can quantify cell migration and avoid the problem of possible cell remnants on the electrode surface after wounding [17,18]. The EF method applies an elevated field to prevent cells from attaching or migrating to the electrode. The surrounding cells outside the cell-free electrode migrate into the open space after turning off the electric fence. Hence, cell migration can be followed and quantified. One of the main advantages of the ECIS EF method is that ECM proteins coated on the electrode remain intact before cells migrate onto them. This function is crucial for investigating the regulation of cell migration by cell–ECM interactions. Another advantage of the EF method is its capability to generate a complete cell-free area that excludes residual bodies’ influence from wounded cells on cell migration.

In this study, we applied the ECIS EF method to investigate HaCaT cell migrations on different substrates, and the subsequent behavior changes of the cells were also clarified. Zeta-potential measurement assessed by an electro-kinetic analyzer was used to measure the surface charges of different substrates. Biochemical assays, such as alamarBlue^®^analysis, correlated with the ECIS data. Herein, we highlight the practicability of the ECIS EF method in assessing cell migration.

## 2. Materials and Methods

### 2.1. Cell Culture

Human keratinocytes (HaCaT cells, provided by Dr. Jehng-Kang Wang, National Defense Medical Center, Taipei, Taiwan) were cultured in Dulbecco’s modified Eagle’s medium with 4.5 gm/L glucose (DMEM-HG, Gibco, Grand Island, NY, USA) and supplemented with 10% fetal bovine serum (Gibco, Grand Island, NY, USA), 100 U/mL penicillin, 100 μg/mL streptomycin (HyClone, Logan, UT, USA), and 1% L-glutamine solution (Corning, New York, NY, USA). HaCaT cells were cultured under 5% CO_2_ humidified condition at 37 °C. The cells were 70% confluent, they were subcultured with standard trypsinization procedure, and the medium was changed twice a week. HaCaT cells were seeded at a density of 8 × 10^4^ cells/well in this study.

### 2.2. Instrumentation

**Figure 1:**
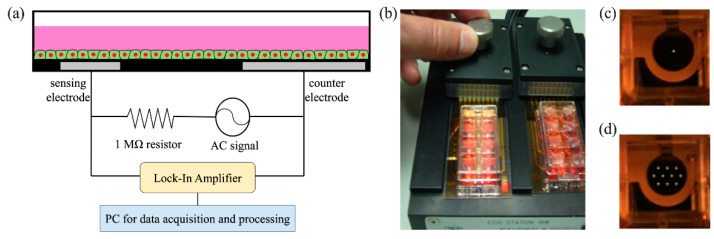
(**a**) Schematic of the ECIS system. (**b**) Image of the ECIS probe station connected with two electrode arrays. Each array contains eight rectangular electrode wells. (**c**) For an 8W1E array, each of the eight wells has one sensing electrode with 250-μm in diameter. (**d**) For an 8W10E array, each of the eight wells has ten identical sensing electrodes with 250-μm in diameter.

Electrode arrays (8W1E and 8W10E), ECIS Zθ instrument, and acquired software for ECIS measurement were obtained from Applied Biophysics (Troy, NY, USA) (Figure 1a). For the electrode array 8W1E and 8W10E, each of the eight wells has a bottom area of 0.8 cm^2^ and contains one and ten small working electrodes with 250-μm diameter, respectively (Figure 1b–d). Before cell inoculation, the electrode arrays were treated with plasma for 1 min, providing an intense cleaning of the electrodes and sterilizing the entire array. In the experiment, we immersed the electrodes with 1 mg/mL peptide solution (poly-L-lysin (PLL, P7890, MW15K-30K, Sigma, St. Louis, MO, USA), poly-D-lysine (PDL, P7886, MW 30K–70K, Sigma, St. Louis, MO, USA), and 200 μg/mL collagen type-I (Collagen, 354236, Corning, New York, NY, USA) for 30 min at room temperature. Afterward, electrodes were thoroughly rinsed with a serum-free medium three times to remove non-adherent protein or peptides.

### 2.3. ECIS Methods

#### 2.3.1. Multiple Frequency Time-Series (MFT) Measurements

After initial cell seeding, MFT measurement was applied to monitor cell attachment and spreading for 24 h while a confluent cell layer was established and lasted until the end. The program measured each well at 11 pre-defined frequencies ranging from 62.5 Hz to 64 kHz. Using ECIS, Wegener et al. reported that measured capacitance at 40 kHz decreases appropriately linearly with cell coverage on the sensing electrode [10]. In addition, it has been reported that 4 kHz is a suitable frequency for ECIS to monitor wound healing migration of HaCaT cells [19]. Thus, in this study, we used capacitance time series at 64 kHz to display dynamic changes in cell attachment and spreading and resistance time series at 4 kHz to show the migratory behavior of HaCaT cells.

#### 2.3.2. Assess Cell Migration Ability with an Electric Fence (EF) Assay

**Figure 2:**
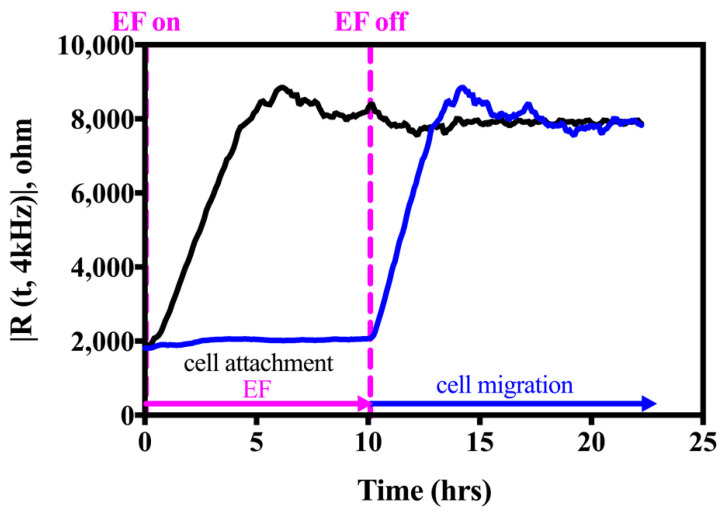
The electric fence function (blue trace) prevents HaCaT cells from attaching and spreading on the ECIS electrode for 10 h. The black trace represents the simultaneous measurement without EF after seeding cells. EF is turned on at the cell inoculation and turned off after the cells form a confluent layer (magenta dashed line). Cells are inoculated at time = 0 h, and impedance measurements were performed by MFT using 11 pre-defined frequencies ranging from 62.5 Hz to 64 kHz. Here, only the resistance time-series data at 4 kHz are shown.

After cells were inoculated into the electrode wells, a series of high current pulses were defined in the ECIS system and applied to prevent cells from attaching and spreading onto the working electrode. Specifically, for 8W1E: 3 successive sinusoidal (pulse shape) pulses, 1 mA at 40 kHz, were applied per 5 min; for 8W10E: 3 successive sinusoidal pulses, 6 mA at 40 kHz, were applied per 5 min. For both cases, the pulse is on for 200 ms (pulse duration) and off for the next 200 ms. The underlying consideration for selecting a suitable pulse frequency is the voltage generated in a cell-free well. To calculate the voltage generated in a cell-free well, check the resistance of the well at 40 kHz. For 8W1E and 8W10E arrays, they are about 2000 and 300 Ohms, respectively. Multiply these numbers by their applied EF currents (1 mA and 6 mA) to obtain the voltages of approximately 2 V. This voltage has given satisfactory results for excluding HaCaT cells attachment to the sensing electrode surface. After a confluent cell layer on the rest of the well was established, the electric fence (EF) was stopped. The cells migrated into the cell-free electrodes, and the cell migration kinetics were measured afterward (Figure 2). The migration speed was calculated with the slope (S) and halfway recovery time from baseline to plateau (T50).

#### 2.3.3. Frequency Scan Measurement

The change of cellular morphology was quantified by measuring the impedance of each electrode before and covered with HaCaT cells at 25 different frequencies, ranging from 31.25 Hz to 100 kHz. By comparing the experimental data of the cell-covered electrode with the calculated values obtained from a suitable cell-electrode model described previously [20], morphological parameters such as junctional resistance between adjacent cells (R_b_), the cell–substrate distance (h), and capacitance of the cell membrane (C_m_) can be obtained.

#### 2.3.4. Micromotion Measurement

The subtle change in cell morphology can be easily detected in a small population of cells. Rapid time collection (RTC) in ECIS can collect those fast oscillatory data and illustrate the micromotions of cells in response to different coating substrates via numerical analysis. For micromotion measurement, impedance data of each well was recorded every second with 4 kHz RTC for 0.6 h until 2048 points had been acquired. After data collection, variance 32 (Var32) was chosen to monitor time-series impedance fluctuations. The 2048 data points were split into 64 groups with 32 points each and divided by the average value. Each group’s variance (the square of standard deviation) was calculated and then averaged for all the sets.

### 2.4. Zeta Potential Analysis

An electro-kinetic analyzer (SurPASS, Anton-Paar GmbH, Austria) was used to measure the surface charges of all samples (10 × 20 mm areas per sample) with the adjustable gap cell immersed in a streaming potential of 10 mM NaCl pH = 7.4 aqueous solution as the electrolyte. The values of the zeta potential (*ζ*) were calculated based on the Helmholtz–Smoluchowski equation:

(1) $$\zeta =\left(\frac{\eta \lambda }{\varepsilon_{0}\varepsilon_{\gamma }}\right)\left(\frac{\Delta E}{\Delta P}\right)$$

where Δ*E* is the variation in the streaming potential, Δ*P* is the pressure difference across the fiber plug, *ε_γ_, η*, and *λ* are the permittivity, viscosity, and conductivity of the electrolyte, and ε_0_ is the vacuum permittivity.

For serum adsorption analysis, all samples were immersed in 10% fetal bovine serum (FBS) solution for 24-h at room temperature and then washed with a buffer for 1 min. Subsequently, the surface charge values of all samples were detected and measured using *ζ*.

### 2.5. Analysis of Initial Adhesion

For assessment of the initial cell adhesion on different substrates, an alamarBlue^®^ cell viability assay was conducted. The cells were seeded in a 24-well plate pre-coated with varying substrates with a density of 10 × 10^4^ cells per well and allowed to attach for 45 min on a rotor at room temperature. After removing the culture medium, 100 μL of alamarBlue^®^ Reagent (G-Bioscience, St. Louis, MO, USA) in an amount equal to 10% of the volume was added to the well and incubated for 4 h. The alamarBlue^®^ reagent was collected into a 96-well plate and the fluorescent intensity measured using a microplate reader with 570 nm light as the excitation source, and emission was measured at 590 nm. The % reduction of alamarBlue^®^ reagent was calculated with the following formula:

(2) $$\%\;\mathrm{of}\;\mathrm{Reduction}\;\mathrm{of}\;\mathrm{alamarBlue}\;\mathrm{reagent}=\left(\frac{\mathrm{Experimental}\;\mathrm{RFU}-\mathrm{Negative}\;\mathrm{Control}\;\mathrm{RFU}}{\mathrm{Positive}\;\mathrm{Control}\;\mathrm{RFU}-\mathrm{Negative}\;\mathrm{Control}\;\mathrm{RFU}}\right)\times 100$$

### 2.6. Statistical Analysis

Experimental evidence was calculated using one-way or two-way ANOVA (Prism 8, GraphPad Inc., San Diego, CA, USA) to determine the statistical significance of the control group. The significance was labeled at a *p*-value less than 0.05. All of the experiments were at least three repetitions, and the data were presented as mean ± standard error of the mean (SEM).

## 3. Results

### 3.1. Effects of Different Substrates on HaCaT Cell Attachment and Spreading

**Figure 3:**
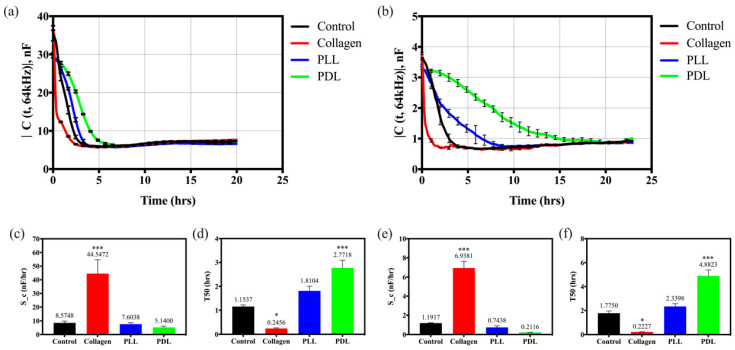
Attachment and spreading of HaCaT cells on different substrates, which are control (bare array, black), collagen (red), PLL (blue), and PDL (green). (**a**,**b**) Capacitance time series of HaCaT cells attached on different coated substrates measured at 64 kHz using 8W10E and 8W1E arrays, respectively. (**c**,**e**) Slope and (**d**,**f**) T50 analysis of the cell adhesion using 8W10E and 8W1E arrays, respectively. Data were presented as mean ± SEM (N > 5 in each group, and the N number is the number of total wells). * *p* < 0.05, *** *p* < 0.001, compared with the control group.

Adhesion analysis of HaCaT cell adhesion seeded on four different substrates is depicted in Figure 3. Two quantities in the extracted data were exploited to describe the cell attachment behavior to quantify cell adhesion ability. One is the average slope of the capacitance shift utilizing the linear regression between cell coverage and capacitance decrease related to time. Averaged slope, S_c=|−ΔC/Δt|, can correspond to the rate of cell spreading between *C* = 3 µF and *C* = 1.5 µF in the single electrode array (8W1E) and *C* = 30 µF and C = 10 µF in the multiple electrode array (8W10E). It avoids obstacles from cells settling in the beginning and locomotion in the plateau phase. Another quantity factor is the time required for the cells to spread out on the half area of the working electrode (T50). Figure 3a,b illustrate the time course of the capacitance measured at 64 kHz when HaCaT cells were seeded into the measuring array in 8W10E and 8W1E, respectively. The averaged slope and T50 of HaCaT cell attachment and spreading on the different coated substrates measured with 8W10E arrays are shown in Figure 3c,d. In the cell attachment and spreading experiments with 8W1E arrays, the slope (Figure 3e) was calculated to be 6.9381 nF/h for collagen, 1.1917 nF/h for control, 0.7438 nF/h for PLL, and 0.2116 nF/h for PDL. The T50 was quantified to 0.2227 h for collagen, 1.7750 h for control, 2.3398 h for PLL, and 4.8823 h for PDL (Figure 3f). The data demonstrate that the HaCaT cells attached and spread significantly faster on collagen-coated substrates than any other substrates examined. Interestingly, poly-lysine groups (PLL and PDL) displayed slower than uncoated (control) electrodes. The attachment and spreading ability of keratinocytes seemed to be inhibited when cells were seeded on both poly-lysine-coated electrodes. Moreover, comparing these two arrays, the slope of the single electrode array has clearer resolution than the multiple electrode arrays.

### 3.2. Effects of Different Substrates on HaCaT Cell Migration

After observing the cell attachment and spreading of the HaCaT cells seeded on the different substrate coated electrodes, cell migration motility was further investigated with the EF technique. Unlike a traditional wounding assay forcing the cells to detach from the electrode before assessing cell migration, EF assay prevents cell attachment and spreading on the defined area by applying short but high current pulses to the working electrode. Resistance is determined by the membranes of cells and their cell–cell and cell–matrix contacts to block the current flow. Since the cell membrane as an insulator would impede current flow and cells moved from outside onto the inner electrode, an increase in the measured resistance of ECIS is seen. Therefore, resistance measurement at lower frequencies can provide information on the barrier integrity and indicate cell migration on the blank electrode. Two exploited quantities are used to determine the migration rate as described above. One is the average slope of the resistance shift (S_r=|ΔR/Δt|), which is calculated by the linear regression of the resistance between cell-free and resistance increase in time and can correspond to the rate of cell migration. Another quantity, T50, is the time required to reach the halfway resistance value from baseline to plateau.

**Figure 4:**
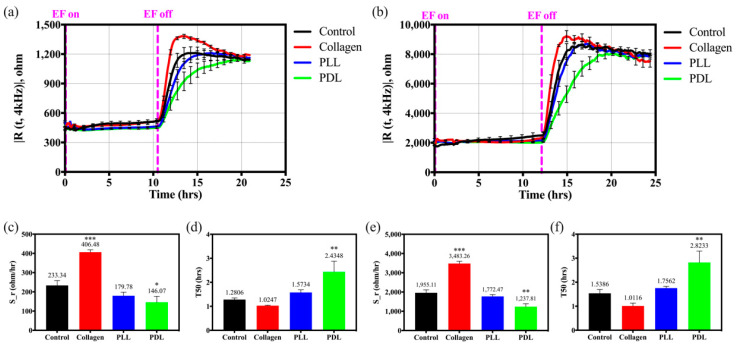
Migration analysis of HaCaT cells seeded on different substrates, which are control (bare array, black), collagen (red), PLL (blue), and PDL (green) with the application of EF. (**a**,**b**) Resistance time series of HaCaT cell migration on different coated substrates measured at 4 kHz using 8W10E and 8W1E arrays, respectively. EF activated and de-activated indicated with the magenta dashed line. (**c**,**e**) Slope and (**d**,**f**) T50 analysis of the cellular migration using 8W10E and 8W1E arrays, respectively. Data were presented as mean ± SEM (N > 5). * *p* < 0.05, ** *p* < 0.01, *** *p* < 0.001, compared with the control group.

Migration analysis of HaCaT cells seeded on different substrates is presented in Figure 4. Figure 4a,b indicate the resistance time series of HaCaT cell migration on different coated substrates measured at 4 kHz in the multiple electrode array and the single electrode array. In Figure 4c,d, we determined the cell migration rate and half-time required for to mean resistance (T50) value of HaCaT cells on different coated electrodes with 8W10E arrays. Similar results performed on 8W1E arrays are illustrated in Figure 4e,f. The slope was 3483.26 ohm/h for collagen, 1955.11 ohm/h for control, 1772.47 ohm/h for PLL and 1237.81 ohm/h for PDL, respectively (Figure 4e). The T50 was quantified to 1.0116 h for collagen, 1.5386 h for control, 1.7562 h for PLL, and 2.8233 h for PDL (Figure 4f). HaCaT cells spread significantly faster on the collagen-coated substrate than on artificial polymers or protein examined on both arrays. PLL and PDL-coated groups seem to inhibit cell migration compared with uncoated groups. Moreover, the single electrode arrays exhibit a better slope resolution than the multiple electrode arrays on cell adhesion and migration analysis. Because the 8W10E arrays provide more cell population than the 8W1E arrays, the overall attachment and spreading ability can be smoothed.

### 3.3. Effects of Morphological Parameters on the HaCaT Cultured on Different Substrates

**Figure 5:**
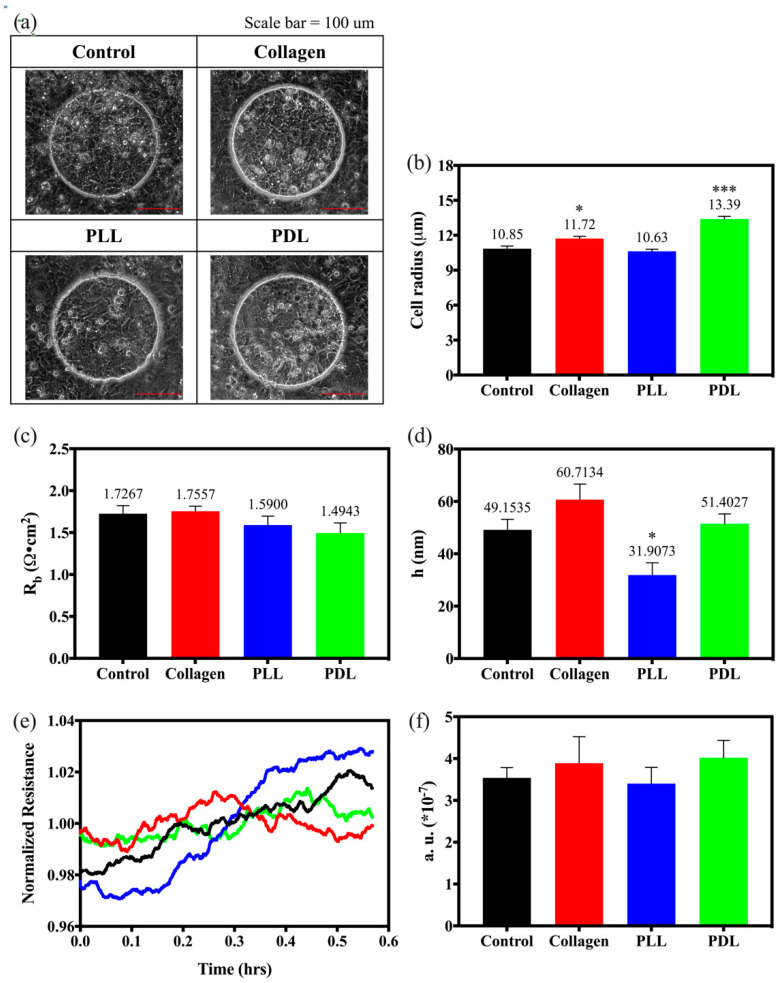
Morphological change and micromotion analysis of HaCaT cells cultured on different substrates, which are control (bare array, black), collagen (red), PLL (blue), and PDL (green). (**a**) Morphology of HaCaT cells cultured on the electrodes with different coated substrates. Scale bar = 100 μm. (**b**) The averaged cell radius (r_c_) is calculated by the morphological images using ImageJ software by the formula, $r_{c}=\sqrt{A_{c}/\pi }\;$. (**c**) The resistance between cells (Rb) and (**d**) cell-substrate distance (h) are obtained by comparing the frequency scan data with the calculated values obtained from the cell-electrode model. Data were presented as mean ± SEM (N = 50 cells in each group for the analysis of cell radius, N > 5 in each group for the ECIS modeling analysis). * *p* < 0.05, *** *p* < 0.001, compared with the control group. (**e**) Normalized resistance time-course measurement after cell adhesion on different substrates, which are control (bare array, black line), collagen (red), PLL (blue), and PDL (green). Each curve consists of 2048 points taken at a 1-s sampling rate and used to calculate its Var32 value. (**f**) Var32 values of the HaCaT cells cultured on different substrates. Data were presented as mean ± SEM (N > 10 in each group).

To further understand the effect of cell attachment on different coated substrates on morphological changes of HaCaT cells, especially on cell–cell and cell–substrate interactions, a frequency scan was recorded from cell-covered electrodes after 24 h of time-course monitoring. Moreover, the optical microscopic images were taken after finishing the frequency scan recording. Figure 5a displays the microscopic images of HaCaT cells attached to the different substrate-coated electrodes. HaCaT cells appear flat and round in similar shape on the uncoated (control) and PLL-coated electrodes. The cells attached to the collagen-coated electrode have almost the same cell size as those attached to the previous two substrates. However, the cells seeded on the collagen-coated electrodes display a relatively stereoscopic view (possess a more significant dimension). In Figure 5b, the averaged cellular radius (*r_c_*) on each coated substrate was calculated from the optically determined average area of cells (*A_c_*) with ImageJ software by the formula $r_{c}=\sqrt{A_{c}/\pi }$. As observed in the microscopic images shown in Figure 5a, HaCaT cells attached to the PDL-coated electrodes have a larger cellular radius of 13.39 μm, following 11.72 μm attached to the collagen coating ones.

After fitting the frequency scan data with the cell-electrode model calculation, the junctional resistance between cells (*R*_b_) and the average cell–substrate separation (*h*) of the control HaCaT cells were 1.7267 Ω∙cm^2^ and 49.1535 nm, respectively. These are typical parameter values for many epithelial or endothelial cell lines. Morphological parameters in response to the different substrates are illustrated in Figure 5c,d. Figure 5c demonstrates that the junctional resistance between cells (*R*_b_) is similar for all the four different substrate-coated electrodes (*p* > 0.05). However, the cell–substrate separation (*h*) on PLL-coated electrodes has the lowest value (31.9073 nm), indicating that the HaCaT cells have a relatively flattened cellular morphology on PLL-coated electrodes than on the other coatings (Figure 5d).

When live cells grow into a confluent layer, cell activity may express itself as a fluctuation in the measured impedance. Vigorous cell motion may imply more fluctuation in ECIS data. To detect rapid changes in impedance, rapid time collection (RTC) was applied after 24 h of cell seeding, and data were used to analyze the micromotion of the HaCaT cells attached to different substrates. In this assay, impedance data were taken every second at 4 kHz for 2048 s, and subtle fluctuations in the resistance time course can be sensitively detected. The normalized data divide each point with its average value before calculating the fluctuations (Figure 5e). Var32 values were calculated to distinguish cell micromotions on different coated groups, as shown in Figure 5f. Compared with control cells, the increased fluctuations (Figure 5e) and Var32 values (Figure 5f) for the cells attached to the collagen and PDL-coated electrodes generally represent increased micromotion. In Figure 5f, cells have higher Var32 values on both PDL-coated (4.02 × 10^−7^) and collagen-coated electrodes (3.89 × 10^−7^) than on the uncoated electrodes (3.54 × 10^−7^), indicating that they have more fluctuations in the time-course monitoring and more intense micromotion. This observation correlates with Figure 5d; PDL-coated and collagen-coated electrodes have a larger cell–substrate separation distance at 51.4027 nm and 60.7134 nm.

### 3.4. Effects of Electrostatic Cell-Substrate Interactions on the Initial Cell Adhesion

**Figure 6:**
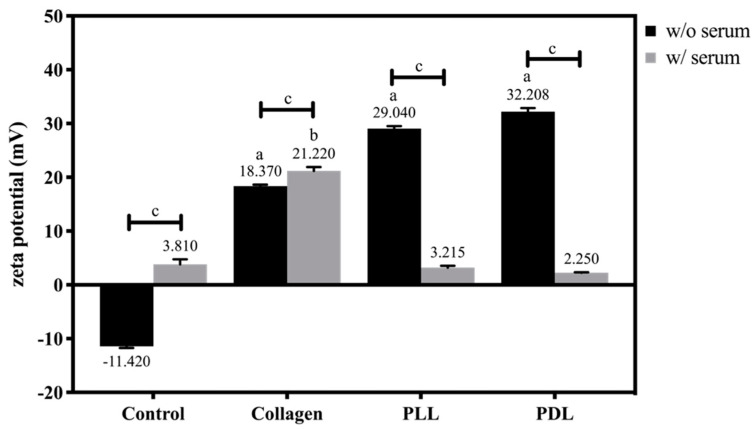
Zeta-potential analysis of different substrates immersed in PBS (w/o serum, black bars) and culture medium (w/ serum, grey bars). Data were presented as mean ± SEM (N = 4). ^a^ *p* < 0.001, compared with the control group in PBS, ^b^ *p* < 0.001, compared with the control group in medium, and ^c^ *p* < 0.05, compared between the groups with or without serum adsorption.

To identify the factors that may affect cell adhesion, surface charge is one of the crucial properties that should be measured. These four different substrates were coated on the microscopic slides in 10 × 20 mm areas, measured with an adjustable gap cell of surPASS under the streaming potential of 10 mM NaCl aqueous solution. Figure 6 shows the zeta potential of the different substrates immersed in PBS (w/o serum) and culture medium (w/serum). Because the glasses were activated with plasma cleaner initially, the surface carried a negative charge (−11.420 mV) in the control group with PBS immersion. Poly-lysine groups as the polycationic peptide, carried with the amino groups hydrolyze in the neutral environment, have a similar zeta potential (~30 mV). In collagen, a kind of zwitterion, the zeta potential is affected by the pH value and exhibits an isoelectric point (IEP) of 9.3. The zeta potential we measured herein was 18.370 mV due to the neutral environment, and made the collagen molecule carry a positive charge. After serum adsorption, the zeta potentials increased to about 3.810 mV and 21.220 mV in both uncoated and collagen-coated groups, suggesting possible adsorption of a small amount of positively charged serum protein. However, the zeta potentials of the PLL and PDL groups decreased after serum absorption, which might be regarded as the adsorption of the negative charge of serum proteins. Overall, the zeta potential was 21.220 mV for collagen, 3.810 mV for control, 3.215 mV for PLL, and 2.250 mV for PDL after serum immersion.

**Figure 7:**
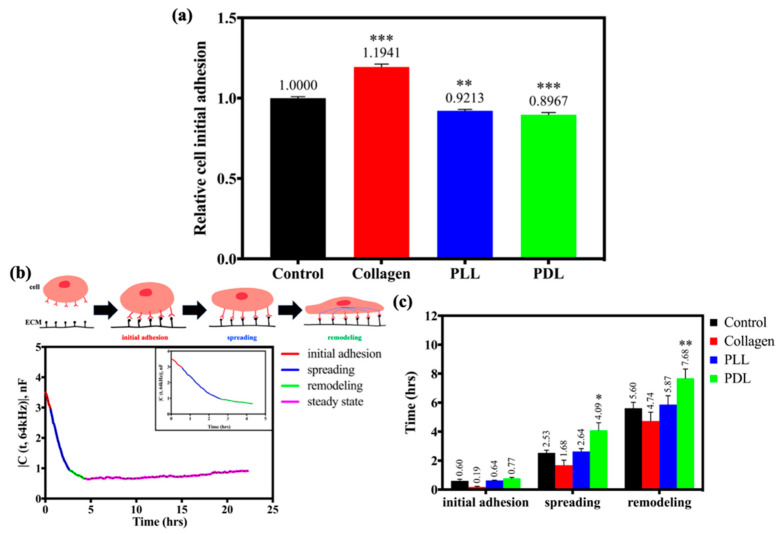
Initial adhesion analysis of HaCaT cells seeded on different substrates. (**a**) Relative cell initial adhesion on different coated substrates, which are control (bare array, black), collagen (red), PLL (blue), and PDL (green), via alamarBlue^®^ cell viability assay. (**b**) Diagrams of the cell adhesion process and the capacitance profile in the time series data measured by ECIS. (**c**) Analysis of HaCaT cell adhesion time on different coated substrates using the ECIS time-course measurement. Data were presented as mean ± SEM (N > 8 for the alamarBlue^®^ cell viability assay, N > 10 for the analysis of cell adhesion time, and the N number is the total wells). * *p* < 0.05, ** *p* < 0.01, *** *p* < 0.001, compared with the control group.

The amount, type, and refolding degree of proteins adsorbed on the surface were determined by the surface charge and potential, influencing the cell adhesion process. To further understand the effect of cell adhesion properties by the surface charge and potential, we used the alamarBlue cell viability assay and adhesion time analysis to determine the HaCaT cell attachment to the coated electrodes. As shown in Figure 7a, the relative initial adhesion of HaCaT cells seeded on the collagen-coated substrate has the highest value (1.1941), followed by the uncoated electrodes (1.0000), PLL-coated electrodes (0.9213), and PDL-coated electrodes (0.8967). Figure 7b reveals the cell attachment model and the analysis methodology in the ECIS time-course measurement. Distinct stages in cellular adhesion can be distinguished from the slope differences in the monitoring procedure: initial adhesion, spreading, remodeling, and the steady-state. Analysis of adhesion time, especially on the initial adhesion, spreading, and remodeling, is revealed in Figure 7c. Overall, the HaCaT cells seeded to the collagen-coated electrodes have a faster attachment speed in these three stages, 0.19 h for initial adhesion, 1.68 h for spreading, and 4.74 h for the remodeling. Furthermore, the PDL-coated electrodes exhibit the longest time for cells to attach in these three stages, indicating that the HaCaT cells attached to PDL-coated substrates require more time than others.

## 4. Discussion

Extracellular matrix (ECM) is an extraordinarily complex and unique meshwork composed of structural proteins and glycosaminoglycans [21]. It is an essential material for cell anchorage and support [22], and its formation is also crucial for wound healing. Collagen is the main structural protein found in the human body and a component of skin tissue beneficial in wound healing [23]. Many synthetic materials serve as good ECM in the modern application of tissue engineering. Poly-lysine is a chemically synthesized cationic polymer to facilitate cell adhesion to the surfaces, and widely used for further investigation [24]. It consists of L-lysine (e.g., PLL) and D-lysine (e.g., PDL), which refer to the chirality at the central carbon [25]. At lower concentrations, poly-lysine promotes cell adhesion and proliferation of MSC in the coating method [26] and indicates that the substrate polycations affect cells via interaction with the anionic site of the cell surface.

There are limited experimental methods to investigate the interaction of the cells with different substrates quantitatively. Consequently, monitoring the attachment and migration of keratinocytes to various developed biomaterials is profitable. This study successfully demonstrated that the EF could be applied to evaluate cell adhesion and migration speed with the different slopes in capacitance and resistance, respectively. Among the substrates, collagen provides the human keratinocytes (HaCaT cells) a better environment to attach and migrate than the conventional cultureware and synthetic poly-lysine. Moreover, we observed that cell adhesion and migration processes were highly determined by the surface charge of the substrates in the culture conditions. Cell migration is an integrated process of many biological functions and pathological conditions, and can be summarized into four steps: membrane extension, attachment formation, contraction, and rear release [27]. Among them, adhesion-related processes play an essential role in cell migration. At proper adhesiveness, the cells can achieve efficiencies from attachment and detachment in cell migration [28].

Suitable materials are widely used to modify traditional tissue cultureware to provide better cell adhesion and migration abilities. However, several intrinsic reasons may affect these behaviors. Firstly, the materials used to coat the cultureware are usually macromolecules (e.g., proteins or polysaccharides) or small molecules (e.g., synthetic polymers). Collagen is the most abundant protein in mammals; moreover, the major component of the skin, which provides human keratinocytes an excellent environment for migration in the wound healing process [29,30]. Figure 3 and Figure 4 demonstrate time-courses of the capacitance and resistance measured at 64 kHz and 4 kHz when keratinocytes are seeded on different coated electrodes. Human keratinocytes seeded on PLL and PDL-coated groups spend more waiting time (1 h) to spread than cells on the collagen-coated substrate in the nascent attachment. The adhesion rate of PDL (0.2116 nF/hr) is clearly lower than collagen-coated (6.9381 nF/hr) and naked electrodes (1.1917 nF/hr), as shown in Figure 3. While keratinocytes are placed on the synthetic polymers, they need to build a provisional matrix in the wound bed to facilitate migration. Keratinocytes presumably require more time to spread by secreting self-produced ECM proteins when attached to the synthetic biomaterials [10]. Our observations support this point of view, as HaCaT cells migrate faster on the collagen-coated surface than on naked electrodes and the other polymer-coated electrodes.

Homochirality is a common property of amino acids and carbohydrates. Many pharmaceuticals and biopolymers in current use are chiral compounds [31]. Although the two enantiomers reveal nearly the same physiochemical properties, they both show a remarkable difference in their biological functions [31]. Although most amino acids can exist in both forms, organic lifeforms are almost exclusively made of L-configuration amino acids [32]. Comparing the effects of chirality of poly-lysine on gene delivery efficiency demonstrates that both polymers show no inhibitory effect on cell proliferation. Still, PLL has higher transfection efficiency than PDL [33]. We can assume that the chirality will affect the viability, attachment, and differentiation of living cells on culture, especially the L-configuration. Interestingly, in comparison with PLL-coated groups, PDL-coated groups significantly reduce cell spreading and cell migration rates in our results. Previous research suggests that the PDL is a suitable coating for the neural cells [34,35]; it improves the attachment and growth of the primary neurons, glial cells, and neuroblastomas. However, more experiments should be conducted to investigate if the neural cells can migrate faster on the PDL-coated electrodes through the EF technique.

The velocity of migrating keratinocytes also appears to be influenced by the nature of the substratum. Previous reports on cell migration have shown that PLL exerts either stimulatory or inhibitory effects depending on the cell type, primarily related to the different capabilities to synthesize ECM-proteins as migration substrates [36]. Furthermore, anionic sites on the cell surface interacting with the substrate cations are related to various cell functions [37]. The most common parameter describing the surface potential of biomaterials in contact with a liquid is the zeta potential (ζ), which is the potential developed at the material–liquid interface. Its precise measurement is crucial for designing biomaterial interfaces [38]. Simultaneously, cell–biomaterial electrostatic interactions are critical in the first phase of cell adhesion [39]. As shown in Figure 6 and Figure 7, the zeta potential was 21.220 mV for collagen, 3.810 mV for control, 3.215 mV for PLL, and 2.250 mV for PDL after serum immersion correlated with the results of initial adhesion analysis. This observation indicates the cell adhesion and migration process were significantly associated with the surface charge of the substrates in the culture conditions.

As the cell membranes impede current flow across the cell layer, an increase in the measured resistance during cell attachment and spreading is seen. While living cells maintain motion on the confluent cell layer, fluctuations in the resistance data reflect the kinetics of cell micromotion and cell viability. Several methods have been proposed to analyze the impedance fluctuations, including variance, power slop, Hurst coefficient, and detrend analysis [40,41]. In Figure 5e, normalized resistance reveals more fluctuations in PDL and collagen groups; however, the averaged Var32 values show no statistical significance (Figure 5f). Additionally, the PLL precursor amino acid occurs naturally; the PDL precursor is an artificial product. PDL is assumed to be resistant to enzymatic degradation and enhances cell adhesion more than the former [36]. The impedance fluctuations are attributed to the greatest change of α and Rb at 4 kHz based on the ECIS model. However, Rb is the resistivity between adjacent cells in a confluent layer, change in h (cell–substrate separation) will significantly affect the measured impedance. PDL-coated groups also demonstrate a larger cellular radius from optical images in Figure 5a,b, higher cell–substrate separation (h) in Figure 5d, and greater fluctuation of cell movement than PLL-coated groups in Figure 5f.

Using the ECIS EF method, we investigated the effects of different surface coatings on HaCaT cell migration. We demonstrated that both adhesion and migration of HaCaT cells in response to different coated substrates are mainly due to the surface charge in the culture condition. The EF method is automated and highly reproducible, similar to the ECIS wounding assay. It usually takes a few hours for the gap area, with a diameter of 250 μm, to fully close. The measured resistance or capacitance time series can be analyzed to quantitate the wound-healing migration. Unlike the ECIS wounding assay, instead of damaging a part of the cell monolayer, the ECIS EF method applies an electrical barrier to each sensing electrode and creates a well-defined exclusion area. After cells outside the sensing electrode reach confluence, the electric fence is switched off and cell migration into the opening is monitored. The advantages of the EF method over mechanical or electrical wounding assays include the intactness of ECM coatings and a complete cell-free area without cell remnants. These advantages make the EF method a unique technique that can investigate the effects of ECM proteins on cell migration for most cell lines.

## 5. Conclusions

This study demonstrated that the EF represents a novel technique that could be applied to evaluate cell adhesion and migration speed without concerns of cell debris on single (8W1E) or multiple (8W10E) electrode arrays in the ECIS system. With the difference in slopes in ECIS monitoring, the cell adhesion and migration abilities can be easily quantified. Among the substrates often used in cell culture modification, collagen provides the human keratinocytes (HaCaT cells) a better environment to attach and migrate than conventional cultureware and synthetic poly-lysine. Furthermore, the surface charge of the substrates plays a crucial role in determining cell adhesion and cell migration in the culture condition. Overall, EF is an excellent alternative method of electric wounding. Still, accurate EF parameters need to be further determined in future studies for its application to various cell types.

## Data Availability

The data presented in this study are available upon reasonable request from the corresponding author.
